# Influence of different standing positions on anatomical parameters of coronal whole‐leg weight‐bearing radiographs in preoperative planning for high tibial osteotomy

**DOI:** 10.1002/jeo2.12085

**Published:** 2024-07-06

**Authors:** Tomoaki Kamiya, Kodai Hamaoka, Akira Ono, Yohei Okada, Makoto Emori, Atsushi Teramoto

**Affiliations:** ^1^ Department of Orthopaedic Surgery Sapporo Medical University School of Medicine Sapporo Hokkaido Japan

**Keywords:** alignment, high tibial osteotomy, hip‐knee‐ankle angle, medial proximal tibial angle, percentage weight‐bearing line

## Abstract

**Purpose:**

The purpose of this study was to assess the differences in lower limb global alignment and anatomical parameters of coronal whole‐leg radiographs, which were generally used in preoperative planning for high tibial osteotomy (HTO), according to different weight‐bearing standing positions.

**Methods:**

Between April 2021 and December 2022, 176 patients (60 males and 116 females) were investigated. Full‐weight‐bearing coronal whole‐leg radiographs were obtained with the patella centred on the femoral condyle. Patients were divided by Kellgren–Lawrence grade (KL‐0, KL‐I, KL‐II and KL‐III) and assessed in two standing positions: legs closed and legs spread. Patients with flexion contractures or those unable to stand with full weight bearing were excluded. The mechanical distal femoral angle, medial proximal tibial angle (MPTA), femorotibial angle, joint line convergence angle, percentage weight‐bearing line (%WBL) and hip‐knee‐ankle angle (HKAA) were measured. The Student's *t* test was used to compare the two standing positions. A *p* value < 0.05 indicated a statistically significant difference.

**Results:**

The MPTAs of legs closed standing and legs spread standing were 84.9 ± 2.6° and 85.1 ± 2.4° in KL‐0, 84.7 ± 2.0° and 84.9 ± 2.1° in KL‐I and 85.0 ± 2.43° and 85.4 ± 2.4° in KL‐II, respectively. There were statistically significant differences in the MPTA between the two standing positions in KL‐0, KL‐I and KL‐II. In contrast, the %WBL and HKAA did not change regardless of the standing position. In the KL‐III group, no statistical significance was observed for any of the anatomical parameters.

**Conclusion:**

Several anatomical parameters were changed between the legs closed standing and the legs spread standing positions. It was suggested that the standing position should be taken into consideration in the planning for HTO.

**Level of Evidence:**

Level IV, Case series with no comparison group.

**Abbreviations**
BMI: body mass index
CT: computed tomography
FTA: femorotibial angle
HKAA: hip‐knee‐ankle angle
HTO: high tibial osteotomy
ICC: intraclass correlation coefficients
JLCA: joint line convergence angle
mLDFA: mechanical distal femoral angle
MPTA: medial proximal tibial angle
OA: osteoarthritis
%WBL: percentage weight‐bearing line

## INTRODUCTION

High tibial osteotomy (HTO) is a procedure that realigns the weight‐bearing line from the medial compartment to the lateral compartment of the knee [25]. By shifting the weight‐bearing axis, the force on the medial knee compartment is reduced [8]. It was reported that achieving the accurate correction angle was a key factor for the long‐term survival of HTO [30]. Coronal whole‐leg radiographs are generally used in the preoperative planning for HTO [21]. Preoperative planning using a picture archiving and communication system or specialised software has a significant effect on the postoperative results of HTO [16, 33]. Therefore, determining the correction angle and gap is one of the most important processes.

In general, the anteroposterior long‐leg view is obtained with the patella centred on the femoral condyles, regardless of the standing or lying position. In patients with preoperative varus knees, the mechanical axis in the single‐leg stance changes to more varus than that in the double‐leg stance [32]. Bardot et al. [1] reported that the joint line convergence angle (JLCA) was also increased with weight‐bearing loading from double‐to single‐leg standing. It was reported that significant differences in the estimated correction angles between the supine and standing radiographs are observed in the planning for HTO [20]. Although weight‐bearing coronal whole‐leg radiographs are essential for preoperative planning, the effect of standing position on anatomical parameters has not been clarified.

The aim of this study was to assess the differences in lower‐limb global alignment and anatomical parameters according to different weight‐bearing standing positions with the legs closed or spread. It was hypothesised that there would be significant differences in anatomical parameters between the two standing positions, which may cause correction errors and clinical failure of HTO. These findings contribute to the surgeon in the preoperative planning of HTO.

## MATERIALS AND METHODS

Digital radiographs were obtained from 176 patients with Kellgren–Lawrence (KL) grade I, II or III varus knee OA [15] or other knee disorders such as meniscus injury and anterior cruciate ligament injury between April 2021 and December 2022. The patients were divided into four groups according to OA severity (KL‐0, KL‐I, KL‐II and KL‐III). A total of 60 males and 116 females were included in this study. Patients with flexion contractures or those unable to stand with full weight bearing were excluded. This investigation was conducted as a case series with no comparison group.

**Figure 1:**
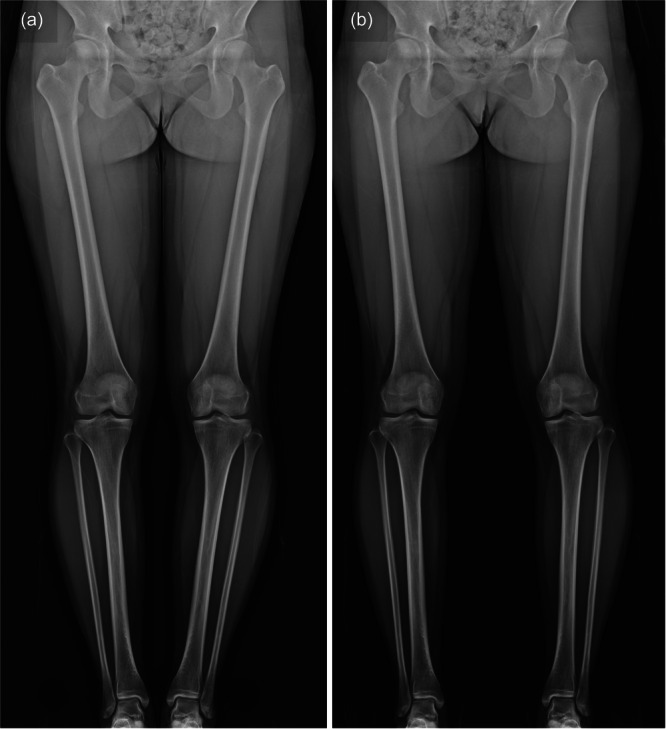
Full weight‐bearing coronal whole‐leg radiographs in the legs closed (a) and legs spread (b) standing with the patella centred on the femoral condyle.

The symptomatic leg was examined, because the evaluation of knee alignment was essential for preoperative planning. Full weight‐bearing coronal whole‐leg radiographs with the patella centred on the femoral condyle were taken for all subjects, including a reference ball (Ø25 mm) for calibration. Each patient was assessed in two standing positions: legs closed and legs spread (Figure 1). In the legs closed standing position, the subjects were indicated to firmly close their feet together. On the other hand, radiographs in the legs spread standing position were taken naturally, with feet shoulder‐width apart. The examinations were performed in a random order.

### Radiographic measurements

The mechanical distal femoral angle (mLDFA), defined as the lateral angle between the femoral mechanical axis and the distal femoral joint line, and the medial proximal tibial angle (MPTA), defined as the medial angle between the tibial mechanical axis and the proximal tibial joint line, were measured on each radiograph. The femorotibial angle (FTA), determined by measuring the angle between the centreline of the femoral and tibial shafts, was also evaluated. The JLCA was defined as the angle made by the two tangential lines between the medial and lateral femoral condyles and the tibial plateau, for which the lateral opening was designated a positive value [19]. The WBL was defined as the line from the centre of the hip to the centre of the ankle. The crossing point of the WBL at the tibial plateau was expressed as a percentage of the total length of the tibial plateau (%WBL), with the most medial edge set at 0% and the lateral edge set at 100%. The hip‐knee‐ankle angle (HKAA) was measured as the angle between the line from the hip centre to the knee centre and the line from the ankle centre to the knee centre; a negative value was defined as varus alignment [4].

All measurements were performed using mediCAD version 5.5.13 modules osteotomy (Hectec GmbH) [27].

### Statistical analysis

All analyses were conducted using EZR (Saitama Medical Center, Jichi Medical University), a graphical user interface for R (The R Foundation for Statistical Computing, version 2.13.0) [13]. Means and standard deviations were determined for each measured anatomical parameter. The demographic data of the four groups were statistically analysed using a one‐way analysis of variance and the chi‐square test. The Student's *t* test was used to compare the two standing positions. A *p* value < 0.05 indicated that the difference was statistically significant. A priori power analysis showed that a minimum of 23 subjects in each group was required to detect differences with a power of 0.8 and an alpha of 0.05. Intraobserver and interobserver reliabilities were assessed using intraclass correlation coefficients (ICCs). Intraobserver reliability was examined at two different times at 2‐week intervals. On the other hand, interobserver reliability was examined by two independent observers. The intraobserver ICC for mLDFA, MPTA, FTA, JLCA, %WBL and HKAA was 0.943, 0.878, 0.996, 0.564, 0.997 and 0.998, respectively. The interobserver ICC for mLDFA, MPTA, FTA, JLCA, %WBL and HKAA was 0.923, 0.871, 0.997, 0.581, 0.997 and 0.995, respectively.

## RESULTS

The mean age at the time of diagnosis was 64.6 ± 17.2 years old (16–92 years old). The demographic data are presented in Table 1. The participants in the KL‐0 group were significantly younger than those in the other groups (*p* < 0.01). Statistically significant differences were observed among the four groups in terms of gender and body mass index (BMI) (*p* < 0.01).

**Table 1:** Demographic data of each group.

	KL grade 0	KL grade I	KL grade II	KL grade III	*p* Value
Age (mean ± SD; years)	41.3 ± 19.4	66.7 ± 11.0	71.2 ± 11.2	75.8 ± 8.6	<0.01
Gender (*n*)					
Male	15 (45.5%)	28 (44.4%)	12 (23.5%)	4 (12.9%)	
Female	18 (54.5%)	35 (55.6%)	39 (76.5%)	27 (87.1%)	<0.01
Height (mean ± SD; cm)	163.3 ± 8.2	160.3 ± 8.9	156.1 ± 9.4	154.2 ± 8.5	<0.01
Body weight (mean ± SD; kg)	64.7 ± 12.0	62.9 ± 13.7	61.9 ± 15.8	62.7 ± 16.5	n.s.
BMI (mean ± SD)	24.2 ± 3.4	24.3 ± 3.7	25.2 ± 5.0	26.2 ± 5.8	n.s.

Abbreviations: BMI, body mass index; KL, Kellgren–Lawrence; SD, standard deviation.

The radiographic parameters of the subjects in the KL‐0 group are shown in Table 2. Statistically significant differences were observed in the MPTA and FTA between the two standing positions (*p* < 0.05). In contrast, the mLDFA, JLCA, %WBL and HKAA did not change regardless of the standing position. In the KL‐I and KL‐II groups, statistical significance was also found for MPTA in the two standing positions (Tables 3 and 4) (*p* < 0.05). In subjects with KL‐III, the radiographic parameters did not change between the two leg positions (Table 5).

**Table 2:** Radiographic parameters of the subjects with KL grade 0 (mean ± SD).

	Leg closed standing	Legs spread standing	*p* Value
mLDFA (°)	86.6 ± 2.3	86.7 ± 2.1	n.s.
MPTA (°)	84.9 ± 2.6	85.1 ± 2.4	<0.05
FTA (°)	176.6 ± 2.5	176.3 ± 2.9	<0.05
JLCA (°)	1.4 ± 1.4	1.3 ± 1.0	n.s.
%BWL	35.0 ± 11.0	35.9 ± 13.0	n.s.
HKAA (°)	−2.7 ± 2.5	−2.6 ± 2.8	n.s.

Abbreviations: FTA, femorotibial angle; HKAA, hip‐knee‐ankle angle; JLCA, joint line convergence angle; KL, Kellgren–Lawrence; mLDFA, mechanical distal femoral angle; MPTA, medial proximal tibial angle; SD, standard deviation; %WBL, percentage weight‐bearing line.

**Table 3:** Radiographic parameters of the subjects with KL grade I (mean ± SD).

	Leg closed standing	Legs spread standing	*p* Value
mLDFA (°)	86.7 ± 2.3	86.9 ± 2.3	<0.05
MPTA (°)	84.7 ± 2.0	84.9 ± 2.1	<0.05
FTA (°)	176.4 ± 3.1	176.2 ± 3.2	n.s.
JLCA (°)	1.5 ± 2.3	1.3 ± 1.1	n.s.
%BWL	36.0 ± 12.3	35.5 ± 12.9	n.s.
HKAA (°)	−2.7 ± 3.0	−2.9 ± 2.9	n.s.

Abbreviations: FTA, femorotibial angle; HKAA, hip‐knee‐ankle angle; JLCA, joint line convergence angle; KL, Kellgren–Lawrence; mLDFA, mechanical distal femoral angle; MPTA, medial proximal tibial angle; SD, standard deviation; %WBL, percentage weight‐bearing line.

**Table 4:** Radiographic parameters of the subjects with KL grade II (mean ± SD).

	Leg closed standing	Legs spread standing	*p* Value
mLDFA (°)	87.0 ± 2.2	87.2 ± 2.2	n.s.
MPTA (°)	85.0 ± 2.4	85.4 ± 2.4	<0.01
FTA (°)	177.4 ± 2.8	177.0 ± 2.9	<0.05
JLCA (°)	1.9 ± 1.0	1.9 ± 1.2	n.s.
%BWL	32.3 ± 10.9	32.9 ± 10.8	n.s.
HKAA (°)	−3.7 ± 3.0	−3.6 ± 2.6	n.s.

Abbreviations: FTA, femorotibial angle; HKAA, hip‐knee‐ankle angle; JLCA, joint line convergence angle; KL, Kellgren–Lawrence; mLDFA, mechanical distal femoral angle; MPTA, medial proximal tibial angle; SD, standard deviation; %WBL, percentage weight‐bearing line.

**Table 5:** Radiographic parameters of patients with KL grade III (mean ± SD).

	Leg closed standing	Legs spread standing	*p* Value
mLDFA (°)	88.3 ± 2.3	88.0 ± 1.5	n.s.
MPTA (°)	83.7 ± 2.1	83.8 ± 2.3	n.s.
FTA (°)	180.9 ± 3.6	180.6 ± 3.9	n.s.
JLCA (°)	3.3 ± 1.8	3.3 ± 1.8	n.s.
%BWL	16.2 ± 13.5	16.8 ± 14.1	n.s.
HKAA (°)	−7.6 ± 3.2	−7.4 ± 3.2	n.s.

Abbreviations: FTA, femorotibial angle; HKAA, hip‐knee‐ankle angle; JLCA, joint line convergence angle; KL, Kellgren–Lawrence; mLDFA, mechanical distal femoral angle; MPTA, medial proximal tibial angle; SD, standard deviation; %WBL, percentage weight‐bearing line.

Changes in the two standing positions are presented in Table 6. The amount of change ranged from 0.5 to 1.0 mm in %BWL and from 0.0° to 0.4° in other parameters. There were no statistically significant differences between the groups. The standard deviation of %WBL was 35.0 in KL‐0, 16.5 in KL‐I, 15.2 in KL‐II and 17.1 in KL‐III, respectively.

**Table 6:** Change between the two standing positions (mean ± SD).

	KL grade 0	KL grade I	KL grade II	KL grade III	*p* Value
mLDFA (°)	0.0 ± 1.1	0.2 ± 0.6	0.2 ± 0.8	−0.3 ± 4.4	n.s.
MPTA (°)	0.3 ± 0.5	0.2 ± 0.8	0.4 ± 0.8	0.2 ± 1.3	n.s.
FTA (°)	0.3 ± 1.1	0.1 ± 0.8	0.3 ± 1.2	0.2 ± 2.2	n.s.
JLCA (°)	−0.2 ± 1.3	0.2 ± 5.2	0.1 ± 0.7	−0.0 ± 1.1	n.s.
%BWL	1.0 ± 35.0	−0.5 ± 16.5	0.6 ± 15.2	0.6 ± 17.1	n.s.
HKAA (°)	0.2 ± 1.6	−0.2 ± 2.2	0.2 ± 0.8	0.2 ± 0.8	n.s.

Abbreviations: FTA, femorotibial angle; HKAA, hip‐knee‐ankle angle; JLCA, joint line convergence angle; KL, Kellgren–Lawrence; mLDFA, mechanical distal femoral angle; MPTA, medial proximal tibial angle; SD, standard deviation; %WBL, percentage weight‐bearing line.

## DISCUSSION

### Radiographic assessments

The most important finding of this study was that the %WBL and HKAA showed no significant change, regardless of the standing position. The centres of the hip and ankle may be unaffected by the standing position. A %WBL of 62%–62.5% is a well‐accepted alignment after HTO because of better cartilage regeneration with favourable clinical outcomes [7, 26, 31]. It was also reported that a significant difference in %WBL was not found between double‐leg and single‐leg stance weight‐bearing conditions [1]. Therefore, ％WBL is considered a useful parameter when preoperative planning is performed for HTO. On the other hand, the mean %WBL was significantly higher in the standing than in the supine radiographs [18]. Furthermore, the standard deviation of the %WBL was relatively large in this study, and the %WBL of some participants was affected by the standing position. These findings should be considered during the preoperative planning for HTO.

The results of this study indicated that the HKAA was also the same in different standing positions. The HKAA is an important parameter for preoperative planning in knee joint surgeries, such as osteotomies and arthroplasty [5, 9]. The correction angles for HTO were determined using Dugdale's or Miniaci's methods [29]. Wang et al. [32] reported that the HKAA in the single‐leg stance of preoperative patients was significantly more varus than that in the double‐leg stance, which was significantly more varus than in the supine position. On the other hand, the difference in the HKAA between standing and supine was small, which might lead to a decreased risk of overestimating postoperative limb alignment [28]. However, approximately 20% of patients had a risk of error in HKAA measurement in two‐dimensional imaging compared with three‐dimensional imaging [18]. In addition, patients post‐HTO showed different patterns of changes [32]. Although the HKAA is constant regardless of standing position in this study, it may not be appropriate for the preoperative planning of knee osteotomy.

In this study, the anatomical parameters of the mLDFA and MPTA were affected by the standing position. It has been reported that the MPTA gradually increases from external to internal rotation relative to the neutral surgical epicondylar axis [14]. In patients with varus grade III or IV on the KL scale, the internal rotation angle was significantly higher in the standing position than in the supine position [6]. The causes of parameter change need to be clarified in future studies.

Changes in the JLCA represent soft tissue correction after HTO [24]. It has been reported that the risk of a greater JLCA decreases after HTO, which is associated with a greater preoperative JLCA in the standing position and the difference between the JLCA in the standing and supine positions [22]. Matsushita et al. [20] reported that the mean JLCA was significantly higher on standing radiographs than on supine radiographs. Furthermore, it has been reported that the JLCA significantly increased from double‐leg to single‐leg stances in patients with isolated medial knee osteoarthritis patients; however, there was no significant difference between the supine position and double‐leg stance for JLCA [1]. Preoperative JLCA of 4° or more were thought to be one of the risk factors for postoperative overcorrection of open wedge HTO [2]. It should be considered that the different standing positions significantly changed the JLCA.

It was reported that the preoperative JLCA on standing position and joint line obliquity could be used to estimate additional alignment changes [12]. Recently, knee deformities have been assessed using three‐dimensional computed tomography (CT). It has been reported that the difference between two‐dimensional and three‐dimensional non‐weight‐bearing JLCA is significant [10]. The relationship between the legs in a closed standing position and the legs spread standing position for coronal alignment using two‐dimensional radiographs was investigated in this study. If weight‐bearing CT becomes common, a three‐dimensional assessment may be essential to evaluate the JLCA.

### Intraobserver and interobserver reliability

The intraobserver and interobserver ICC were relatively high in this study. Nerhus et al. [23] also reported that all ICC values except for JLCA were above 0.80 using the same measurement software of this study. The manual planning for HTO had a higher risk of outliers in deformity angle measurements [17]. Because the accuracy of the preoperative planning was important, it was suggested to use the semi‐automatic software for HTO.

### Limitations

This study has some limitations. First, all patients with KL‐0 complained of knee disorders; therefore, a normal knee may exhibit a different radiographic pattern. However, the aim of this study was to determine the variance in radiographic parameters in the standing position in the planning of knee osteotomy. Therefore, the results of this study may be helpful for knee surgeons. Next, we investigated only the coronal alignment. However, the plain anteroposterior lower leg view is the most popular image for preoperative planning of HTO. In this study, radiographs of the patella centred on the femoral condyles with full weight bearing were obtained. It was suggested that the %WBL and HKAA are applicable when HTO is performed. Third, only six parameters were investigated using measuring software in this study. Other parameters may need to be investigated for preoperative planning. Fourth, this study did not investigate postoperative patients. The coronal and sagittal alignments can change after HTO, and it was not determined whether the %WBL and HKAA were the same in different standing positions.

### Future directions

Recently, it was reported that the external frontal plane lever arm of the knee adduction moment was also one of the useful parameters for HTO [3]. Other parameters in weight‐bearing coronal whole‐leg radiographs might be analysed in the future. Owing to the numerous variations in femoral and tibial torsion, images do not guarantee neutral rotation or complete extension. An average 10° of internal rotation or 20° external rotation in combination with 15° flexion is required to alter the mechanical leg axis measurements by more than 3° [11]. In future studies, three‐dimensional evaluation may be essential.

## CONCLUSIONS

We investigated the differences in lower limb coronal alignment in different weight‐bearing standing positions. The %WBL and HKAA of the subjects with KL‐0, KL‐I, KL‐II and KL‐III were the same in different standing positions. However, mLDFA, MPTA, FTA and JLCA significantly changed between the legs closed standing and legs spread standing positions. It was suggested that the standing position should be taken into consideration in the planning for HTO.

## AUTHOR CONTRIBUTIONS

Tomoaki Kamiya and Kodai Hamaoka designed the study protocol and collected data. Akira Ono assisted with data interpretation. Yohei Okada assisted with statistical analysis. Makoto Emori and Atsushi Teramoto helped to design the study and critically reviewed the manuscript. All authors approved the final manuscript.

## CONFLICT OF INTEREST STATEMENT

The authors declare no conflict of interest.

## ETHICS STATEMENT

This study protocol was reviewed and approved by the Ethical Board of Chitose City Hospital (approval number 2022‐005). All patients gave their informed consent for their participation in and the publication of the study.

## Data Availability

The data that support the findings of this study are openly available.
